# The role of immunotherapy in resectable non-small-cell lung cancer

**DOI:** 10.1177/17588359251361883

**Published:** 2025-08-22

**Authors:** Conor D. Moloney, Patrick M. Forde

**Affiliations:** Beaumont RCSI Cancer Centre, Dublin, V09 V2N0, Ireland; Trinity St. James’s Cancer Institute, Trinity College Dublin, Dublin, D08 XF38, Ireland

**Keywords:** adjuvant therapy, immunotherapy, neoadjuvant therapy, non-small cell lung cancer (NSCLC), peri-operative therapy, surgery

## Abstract

Non-small-cell lung cancer (NSCLC) accounts for 80%–85% of all lung cancer cases, being the leading cause of cancer-related mortality worldwide. Historically, outcomes for patients with resectable disease have trailed those with other solid organ malignancies. Advances in treatment strategies, particularly in immunotherapy (IO), have revolutionised the landscape of lung cancer care. In resectable NSCLC (rNSCLC), including stage III disease, the integration of immunotherapy is increasingly being explored for its potential to reduce recurrences and improve survival outcomes. Several landmark clinical trials have resulted in regulatory approvals, and the rapid adoption of immunotherapy in the neoadjuvant, perioperative and adjuvant settings. This review will comprehensively examine the evolving role of immunotherapy in rNSCLC, with a focus on trial evidence, mechanisms of action, biomarkers and challenges in clinical implementation. We also discuss its implications for multimodal therapy across neoadjuvant, perioperative and adjuvant settings while highlighting potential future directions and identifying unanswered questions.

## Introduction

With an estimated 2.5 million new cases and 1.8 million deaths each year, lung cancer is a major global health burden.^1^ Non-small-cell lung cancer (NSCLC) constitutes 80%–85% of lung cancers. Around 60% of new cases are diagnosed in advanced stages of disease, thus unsuitable for curative-intent therapy.^2^ For the subgroup of patients with early-stage and locally advanced resectable NSCLC (rNSCLC), surgical resection is the cornerstone of curative-intent therapy. However, recurrence rates with surgery alone are high, particularly in stage III disease, where local and distant metastases pose substantial challenges.^3^

Historically, adjuvant (or neoadjuvant) chemotherapy has been used to address micrometastatic disease and improve survival. Yet, adjuvant platinum doublet chemotherapy alone yields a modest survival benefit, approximately a 5% increase in 5-year overall survival (OS),^4^ necessitating the exploration of novel approaches.

Immunotherapy (IO), particularly immune checkpoint inhibitors (ICIs) targeting programmed death-1 (PD-1), programmed death-ligand 1 (PD-L1) and cytotoxic T-lymphocyte-associated protein 4, has shown remarkable efficacy in advanced NSCLC, as monotherapy and in combination with platinum-based chemotherapy.^5–8^ In recent years, the potential of immunotherapy in earlier stage, rNSCLC has garnered attention. Neoadjuvant, perioperative (pre- and post-surgical therapy) and adjuvant immunotherapy have emerged as promising strategies to enhance pathologic responses, eradicate micrometastatic disease, prevent recurrence and ultimately improve OS. This review examines the role of immunotherapy in rNSCLC, with a focus on clinical evidence, biomarkers and practical considerations.

## Defining rNSCLC

**Figure 1.**
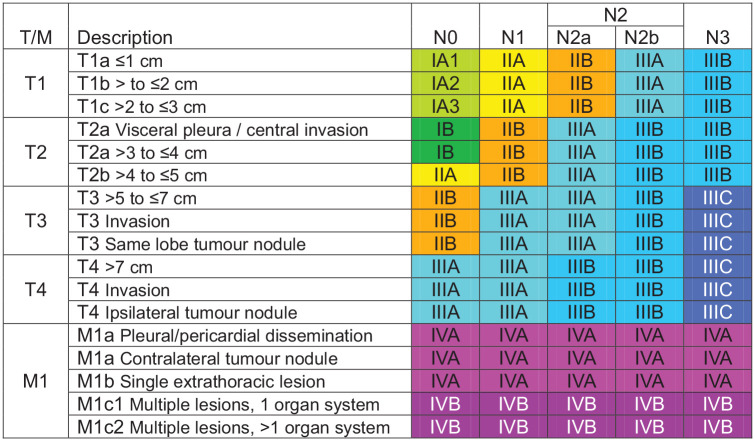
Ninth edition TNM categories. Source: Reprinted from Rami-Porta et al.,^10^ Copyright (2024), with permission from Elsevier. TNM, Tumour-Node-Metastasis.

rNSCLC refers to tumours that can be surgically removed with curative intent. The definition of resectability is largely guided by the TNM (Tumour-Node-Metastasis) staging system. Since 1996, this has been developed by the International Association for the Study of Lung Cancer (IASLC), in collaboration with the American Joint Committee on Cancer and the Union for International Cancer Control. The ninth edition TNM classification of lung cancer took effect in January 2025, and is summarised in figure 1.^9^

Early-stage disease (stage I and II) and select cases of locally advanced (stage III) disease are considered technically resectable. In very early-stage (IA1–IA2) disease, the focus of treatment is primarily on optimising extent of resection and minimising morbidity with minimally invasive surgical techniques. Though surgery remains the cornerstone of curative treatment for resectable stage IB–IIIA/B, the increased risk of recurrent disease necessitates a multimodal approach.

Resectability in stage III disease represents a key area of controversy, as it can be highly individualised, based on factors such as:

1. Tumour size and location (technical resectability) – tumours confined to a single lobe or lung segment are generally considered resectable, while involvement of vital structures such as the trachea or great vessels may preclude surgery.
2. Lymph node involvement – Nodal involvement (N1, single station N2, select multi-station N2) may be technically resectable, with combined modality approaches. Extensive nodal involvement (bulky multi-station N2, or N3) is generally considered unresectable; however, viewpoints on this are evolving.
3. Patient fitness (also known as medical resectability) – Increased age, advanced COPD (chronic obstructive pulmonary disease)/emphysema, poor performance status, impaired pulmonary function (markedly reduced FEV1 (forced expiratory volume in 1 second), DLCO (diffusing capacity for carbon monoxide), lung volume) are all associated with poorer outcomes post-operatively, particularly where a pneumonectomy is required.^11^ Non-respiratory comorbidities, such as cardiovascular disease, diabetes, renal impairment and frailty, should also be considered when deciding on a patient’s fitness for surgery.
4. Local expertise – post-operative surgical outcomes, importantly mortality, are correlated with volume of surgical procedures. As such, it is generally recommended that where a pneumonectomy or tracheal sleeve pneumonectomy will be required for radical resection, they should be performed in high-volume centres.^12^

An international survey initiated by the EORTC-Lung Cancer Group did not reach an agreement on the resectability of smaller tumours with multi-station N2, or larger tumours with single-station N2, highlighting the difficulty on defining resectability for these patients.^13^ For patients with tumours that are felt to be borderline resectable, neoadjuvant or perioperative chemo-immunotherapy may offer the potential to downstage tumours and expand the scope of resectability.

## Systemic therapy prior to immunotherapy

Pre-ICI, the standard of care for resected stage II–IIIA NSCLC, was established by the International Adjuvant Lung Cancer Trial, which demonstrated a 4.1% absolute benefit for OS at 5 years (hazard ratio (HR) for death 0.86, 95% confidence interval (CI): 0.76–0.98) for patients receiving cisplatin-based doublet chemotherapy.^14^ These results were then supported by the LACE meta-analysis, which pooled data from five adjuvant trials (*n* = 4584) and revealed an overall HR of death of 0.89 (95% CI: 0.82–0.96), corresponding to a 5-year absolute OS benefit of 5.4%, for patients receiving platinum-doublet chemotherapy.^4^

The CALGB 9633 study^15^ demonstrated that among patients with resected stage IB NSCLC, a significant survival benefit was only seen in patients with tumours ⩾4 cm (HR 0.69, 95% CI: 0.48–0.99, *p* = 0.043). This set the foundation for the inclusion of only stage IB ⩾4 cm in subsequent adjuvant/neoadjuvant/perioperative immunotherapy trials.

Neoadjuvant versus adjuvant platinum-doublet chemotherapy was compared in the phase III NATCH trial, published in 2010. Patients with stage IA–IIIA rNSCLC were randomised to surgery alone, neoadjuvant carboplatin-paclitaxel for 3 cycles followed by surgery or surgery followed by 3 cycles of adjuvant carboplatin-paclitaxel. There was no statistical difference in 5-year OS, but the patients in the neoadjuvant arm were more likely to receive the planned systemic therapy (97% vs 66.2%).^16^

Neoadjuvant and adjuvant platinum-based chemotherapy can both, therefore, be considered options for patients with rNSCLC.

## Immunotherapy in rNSCLC

### Neoadjuvant setting

The rationale for neoadjuvant systemic therapy is strong. When systemic therapies are delivered preoperatively, patients are generally more able to tolerate and complete the planned therapies, micrometastatic disease can be addressed at an earlier timepoint, and it allows for treatment response assessment prior to surgical intervention.^17^

Due to the difference in mechanism of action compared to chemotherapy, there is further rationale for immunotherapy in the neoadjuvant setting. Data suggest that administering ICIs while the primary tumour and draining regional lymph nodes are in situ results in stronger and more sustained immune responses. This may be driven by increased numbers of tumour neoantigens present prior to resection and represents a unique additional benefit to ICIs.^18,19^

### Monotherapy

Trials evaluating ICI without chemotherapy in the neoadjuvant setting are summarised in Table 1.

**Table 1.** Trials of neoadjuvant immunotherapy alone in resectable stage III NSCLC.

Trial	ICI	Phase	Number of participants	Stages	MPR/pCR	Survival endpoints
Forde et al.^19^	Nivolumab	I/II	21 20 resected	I–IIIA	MPR: 45% (9/20) pCR: 15% (3/20)	5 years OS: 80% (16/20) 5 years RFS: 60% (12/20)
CheckMate 816 (arm 3) Awad et al.^20^	Nivolumab + Ipilimumab	III	221 165 resected	IB–IIIA	MPR: 28.3% vs 14.8% pCR 20.4% vs 4.6%	3 years EFS: 56% vs 44% (HR 0.77) 3 years OS: 73% vs 61% (HR 0.73)
Bott et al.^21^	Nivolumab	I	22 20 resected	I–IIIA	MPR: 45% (9/20)	–
Reuss et al.^22^	Nivolumab + Ipilimumab	Ib/II	9 6 resected	IB–IIIA	pCR: 33% (2/6)	–
NEOSTAR Cascone et al.^23^	(1) Nivolumab vs (2) Nivolumab + Ipilimumab	II	44 33 resected	I–IIIA	(1) MPR: 24% (5/21) pCR: 10% (2/21) (2) MPR: 50% (8/16) pCR: 38% (6/16)	30 months EFS: (1) 82.6% (19/23) (2) 76.2% (16/21)
NeoCOAST Cascone et al.^24^	(1) Durvalumab (2) Durvalumab + Oleclumab (3) Durvalumab + Monalizumab (4) Durvalumab + Danvatirsen	II	84 76 resected	IA3–IIIA	(1) MPR: 11.1% (3/27) pCR: 3.7% (1/27) (2) MPR: 19.0% (4/21) pCR: 9.5% (2/21) (3) MPR: 30.0% (6/20) pCR: 10.0% (2/20) (4) MPR: 31.3% (5/16) pCR: 12.5% (2/16)	–
IoNESCO Wislez et al.^25^	Durvalumab	II	46 43 resected	IB–IIIA	MPR: 19% (8/43)	12 months DFS: MPR 100% Non-MPR 11%
PRINCEPS Besse et al.^26^	Atezolizumab	II	30 30 resected	IA–IIIA	MPR: 0% pCR: 0%	–
LCMC3 Chaft et al.^27^	Atezolizumab	II	181 159 resected	IB–IIIB	MPR: 20% (29/143) pCR: 6% (8/143)	3 years OS: 80% 3 years DFS: 72%e
TOP 1501 Tong et al.^28^	Pembrolizumab	II	35 25 resected	IB–IIIA	MPR: 28% (7/25)	–
NEOMUN Eichhorn et al.^29^	Pembrolizumab	II	15 15 resected	II–IIIA	MPR: 27% (4/15)	–
Gao et al.^30,31^	Sintilimab	Ib	40 37 resected	IA–IIIB	MPR: 40.5% (15/37) pCR: 16.2% (6/37)	5 years OS: 80.4% 5 years DFS: 65.7%

DFS, disease-free survival; EFS, event-free survival; HR, hazard ratio; ICI, immune checkpoint inhibitor; MPR, major pathological response; NSCLC, non-small-cell lung cancer; OS, overall survival; pCR, patological complete response; RFS, recurrence-free survival.

Initial investigations into neoadjuvant immunotherapy focused on ICI monotherapy, largely to assess feasibility, safety and early efficacy signals. In 2018, the phase I/II trial by Forde et al.^19^ established proof of concept of this approach, demonstrating that 2 cycles of neoadjuvant nivolumab (3 mg/kg every 2 weeks) in untreated, resectable NSCLC led to a major pathological response (MPR) in 45% of resected tumours, without delaying surgery or increasing perioperative morbidity. Responses were noted in PD-L1-positive and PD-L1-negative tumours. This was the first study to show that neoadjuvant immunotherapy was safe and feasible in NSCLC and laid a foundation for further trials to build on. Long-term follow-up revealed promising survival outcomes, with 5-year OS and recurrence-free survival (RFS) rates of 80% and 60%, respectively. Of those patients with a tumour MPR, 8 of 9 (89%) were alive and disease-free.^32^ The small sample size and single-arm design, however, prevented direct comparisons with standard treatments.

Subsequent trials have explored both single and dual checkpoint blockade. In NEOSTAR, the combination of nivolumab and ipilimumab resulted in higher MPR and pCR rates than nivolumab alone (50% vs 24% and 38% vs 10%, respectively), and enhanced immune cell population, supporting dual checkpoint blockade as a viable strategy. Despite these encouraging results, the small phase Ib/II study by Reuss et al.^22^ raised caution, as a similar regimen was terminated early due to toxicity, highlighting a potential narrow therapeutic window for dual immunotherapy in the surgical setting.

The CheckMate 816 trial, while primarily designed to assess chemoimmunotherapy, included a third arm evaluating nivolumab plus ipilimumab versus chemotherapy, making it the largest and only phase III trial of neoadjuvant ICI-alone to date.^20^ In this exploratory cohort, dual checkpoint blockade improved MPR (28.3% vs 14.8%) and pCR (20.4% vs 4.6%) compared with chemotherapy alone. However, EFS curves crossed early, and although there is a numerical difference in EFS at 3 years (56% vs 44%), this is not statistically significant. The early divergence in EFS, favouring chemotherapy initially, and a greater number of patients with disease progression before surgery (18 vs 9) suggest that immunotherapy alone may be insufficient to prevent early progression in certain patients, a critical consideration in rNSCLC where cure is the goal.

The NeoCOAST trial explored the addition of novel agents to durvalumab in a platform design. Though limited to a single cycle of neoadjuvant therapy, combinations with oleclumab (anti-CD73), monalizumab (anti-NKG2A) or danvatirsen (anti-STAT3 antisense oligonucleotide) improved MPR rates compared to durvalumab monotherapy (19%–31% vs 11%), with signals of activity even in PD-L1-negative tumours.^24^ These signals align with the phase II COAST trial, in which a survival benefit in unresectable stage III NSCLC was seen in the durvalumab + oleclumab and durvalumab + monalizumab arms, irrespective of PD-L1 status,^33^ suggesting potential avenues for PD-L1-independent response.

Overall, while neoadjuvant ICI monotherapy or dual ICI appears feasible and safe, with the potential for durable responses in select patients, response rates are lower and more variable than those observed in chemoimmunotherapy, which will be discussed next. The absence of a significant EFS or OS advantage in the largest phase III trial (though it was stopped early) reinforces chemotherapy as a therapeutic backbone for now. These findings also underscore the need for biomarkers beyond PD-L1 to guide patient selection.

### Combination with chemotherapy

Trials evaluating chemo-IO combinations in the neoadjuvant setting are summarised in Table 2.

**Table 2.** Trials of neoadjuvant chemoimmunotherapy in resectable stage III NSCLC.

Trial	ICI	Phase	Number of participants	Stages	MPR/pCR	Survival endpoints
CheckMate 816 Forde et al.^34^	Nivolumab vs placebo	III	358 283 resected	IB–IIIA	pCR: 24.0% vs 2.2%	mEFS 31.6 vs 20.8 months (HR 0.63)
NeoTPD01 Zhao et al.^35^	Toripalimab	II	33 30 resected	IIIA–IIIB	MPR: 66.7% (20/30) pCR: 50% (15/30)	–
TD-FOREKNOW Lei et al.^36^	Camrelizumab vs placebo	II	88 82 resected	IIIA–IIIB	MPR: 65.1% vs 15.6% pCR: 32.6% vs 8.9%	24 months EFS; 76.9% vs 67.6% (HR 0.52)
SAKK 16/14 Rothschild et al.^37^	Durvalumab	II	67 55 resected	IIIA(N2)	MPR: 62% (34/55) pCR: 18% (10/55)	24 months OS: 83% 24 months EFS: 68%
Shu et al.^38^	Atezolizumab	II	30 29 resected	IB–IIIA	MPR: 57% (17/30)	–
Zhang et al.^39^	Sintilimab	II	50 30 resected	IIIA	MPR: 43.3% (13/30) pCR: 20% (6/30)	12 months OS: 93.7% 12 months DFS: 85.3%
Neo-Pre-IC Sun et al.^40^	Sintilimab	II	30 20 resected	IIIA/IIIB	MPR: 65% (13/20) pCR: 40% (8/20)	24 months DFS: 75% (surgical group)

DFS, disease-free survival; HR, hazard ratio; ICI, immune checkpoint inhibitor; MPR, major pathological response; NSCLC, non-small-cell lung cancer; OS, overall survival; RFS, recurrence-free survival.

The largest trial to assess neoadjuvant chemoimmunotherapy is the phase III CheckMate 816 trial (*n* = 358). Patients with stage IB–IIIA rNSCLC, and no known EGFR (epidermal growth factor receptor)/ALK (anaplastic lymphoma kinase) alterations, were randomised to receive 3 cycles of nivolumab plus platinum-based chemotherapy or platinum-based chemotherapy alone, followed by surgery.^34^ The addition of nivolumab resulted in a significant improvement in pCR (24% vs 2.2%) and EFS (median 31.6 vs 20.8 months) with the addition of nivolumab. The regimen was well tolerated, with no significant delay in surgery. In a final 5-year update, published in 2025, a statistically significant improvement in OS was confirmed for the nivolumab + chemotherapy arm (5-year OS 65.4% vs 55.0%, HR 0.72, 95% CI: 0.523–0.998),^41^ establishing it as the only neoadjuvant-alone regimen to do so to date. At present, the CheckMate 816 regimen has received regulatory approval, among others, from the European Medicines Agency (EMA; for patients with tumour PD-L1 ⩾1%) and the Food and Drug Association (FDA) in the United States (irrespective of tumour PD-L1 expression).

Several smaller, phase II studies have supported the efficacy of neoadjuvant chemoimmunotherapy combinations, although their generalisability is limited by small sample sizes and shorter follow-up durations. In NeoTPD01, toripalimab plus chemotherapy led to a pCR rate of 50%, with MPR in two-thirds of patients.^35^ Similarly, the TD-FOREKNOW trial showed a marked increase in MPR (65.1% vs 15.6%) and improved 2-year EFS (76.9% vs 67.6%; HR 0.52) with camrelizumab versus placebo.^36^

The SAKK 16/14 trial evaluated neoadjuvant chemotherapy followed by durvalumab in patients with stage IIIA(N2) NSCLC. Although not strictly a concurrent regimen, the sequential strategy yielded an MPR of 62% and a 2-year OS of 83%. These results, while promising, highlight a recurring challenge in this space: the heterogeneity in trial design, endpoints and patient populations, limiting the ability to draw definitive cross-study comparisons.

Taken together, current evidence supports neoadjuvant chemoimmunotherapy as an effective and generally safe approach in rNSCLC, with CheckMate 816 providing the most mature data.

### Adjuvant setting

Immunotherapy trials in the adjuvant setting have tended to evaluate sequential adjuvant platinum-doublet chemotherapy followed by immunotherapy. Some ongoing trials, such as ACCIO^42^ (summarised in Table 3) are evaluating concurrent chemo-immunotherapy versus sequential chemo-immunotherapy and standard of care adjuvant chemotherapy.

**Table 3.** Trials of adjuvant immunotherapy (±chemotherapy) in resectable stage III NSCLC.

Trial	ICI	Phase	Number of participants	Stages	Survival endpoints
IMpower010 Felip et al.^43^	Atezolizumab vs BSC (after adjuvant chemotherapy)	III	1005	IB–IIIA	mDFS (ITT): 65.6 vs 47.8 months (HR 0.85, CI 0.71–1.01) 5 years OS (PD-L1 >50%): 82.7% vs 65.3% (HR 0.47)
PEARLS-KEYNOTE-091 O’Brien et al.^44^	Pembrolizumab vs placebo (± after adjuvant chemotherapy)	III	1177	IB–IIIA	mDFS: 53.6 vs 42.0 months (HR 0.76, CI 0.63–0.91)
CCTG BR.23 Goss et al.^45^	Durvalumab vs placebo (± after adjuvant chemotherapy)	III	1219	IB–IIIA	mDFS (ITT): 60 vs 54 months (HR 0.89, CI 0.75–1.07) mDFS (PD-L1 ⩾25%): 70 vs 60 months, CI 0.71–1.25)
ALCHEMIST chemo-IO (ACCIO) Sands et al.^42^	Pembrolizumab Arm 1: concurrent chemo-IO Arm 2: Sequential chemo followed by IO Arm 3: SOC platinum-doublet chemo	III	Recruiting Est. 1210	IB–IIIA	In progress
ANVIL Chaft et al.^46^	Nivolumab vs surveillance (after adjuvant chemotherapy)	III	Recruitment closed est. 903	IB–IIIA	In progress

BSC, best supportive care; CI, confidence interval; DFS, disease-free survival; HR, hazard ratio; ICI, immune checkpoint inhibitor; IO, immunotherapy; ITT, intention-to-treat; NSCLC, non-small-cell lung cancer; OS, overall survival; PD-L1, programmed death-ligand 1; SOC, standard of care.

IMpower010 was the first phase III adjuvant immunotherapy trial to read out, publishing results in 2021. Patients, including those with ALK/EGFR alterations, with resected stage IB–IIIA NSCLC were randomised to receive 1 year of atezolizumab or best supportive care after adjuvant platinum-doublet chemotherapy.^43^ There was a disease-free survival (DFS) advantage in the intention-to-treat (ITT) population (NE [non-estimable] vs 37.2 months, HR 0.81, 95% CI: 0.67–0.99). The largest benefit was seen in patients with PD-L1 expression >50% (HR 0.43, 95% CI: 0.27–0.68), while those with PD-L1 <1% did not seem to benefit (HR 0.97, 95% CI: 0.72–1.31). OS data remain immature, though in the second OS interim results there is a trend towards an OS benefit in the PD-L1 ⩾1% and ⩾50% populations.^47^ Grade ⩾3 adverse events occurred in 11% of patients, with no new safety signals noted.

The PEARLS-KEYNOTE-091 trial, published in 2022, was a phase III trial of 1 year of adjuvant pembrolizumab.^44^ Unlike IMpower010, adjuvant chemotherapy was optional. In the overall population, there was a benefit for DFS in the pembrolizumab group (53.6 vs 42.0 months, HR 0.76, 95% CI: 0.63–0.91). Interestingly, this time the PD-L1 1%–49% population appeared to benefit the most (HR 0.67, 95% CI: 0.48–0.92). Patients with EGFR alterations also benefited (HR 0.44, 95% CI: 0.23–0.84), though the very small sample size (*n* = 73) precludes definitive interpretation. Grade ⩾3 adverse events occurred in 34% of patients in the pembrolizumab arm, compared to 26% in the placebo arm.

Both trials have influenced regulatory approvals. In the United States, the FDA has licensed atezolizumab for patients with PD-L1 >1%, and pembrolizumab regardless of PD-L1 status. In Europe, the EMA has licensed atezolizumab for patients with PD-L1 ⩾50% and no EGFR/ALK alterations, and pembrolizumab for patients at high risk of recurrence following adjuvant platinum-doublet chemotherapy.

Not all trials have yielded positive results. The Canadian Cancer Trials Group (CCTG) BR.31 trial, a large phase III study of durvalumab versus placebo, failed to meet its primary endpoint.^45^ With a median follow-up of 60 months, no significant improvement in DFS was observed in either the overall population (mDFS 60 vs 54 months, HR 0.89; 95% CI: 0.75–1.07, *p* = 0.21), or the PD-L1 ⩾25% group (mDFS 70 vs 60 months, HR 0.94; 95% CI: 0.71–1.25, *p* = 0.64). These findings raise important questions about whether the efficacy of adjuvant immunotherapy is ICI-specific, or whether patient selection and trial design are contributing factors, as adjuvant chemotherapy was optional in BR.23.

Although adjuvant immunotherapy has yielded promising results to date, the evidence from neoadjuvant and perioperative trials, discussed below, is quite compelling to suggest that introducing immunotherapy pre-surgery is superior. While no head-to-head trials have yet compared neoadjuvant and adjuvant immunotherapy, the consistency and magnitude of benefit observed in neoadjuvant trials, alongside the immunologic rationale, have driven a shift away from adjuvant-only immunotherapy, particularly in resectable high-risk NSCLC.

### Perioperative setting

Perioperative therapy – administered both before and after surgery – has gained increasing traction in rNSCLC. This ‘sandwich’ approach aims to leverage the immune-priming potential of neoadjuvant treatment and consolidate benefit through adjuvant immunotherapy. Table 4 provides a summary of phase III trials of perioperative immunotherapy in rNSCLC.

**Table 4.** Phase III trials of perioperative immunotherapy in resectable NSCLC.

Trial	ICI	Phase	Number of participants	Stages	MPR/pCR	Survival endpoints
KEYNOTE-671 Wakelee et al.^48^	Pembrolizumab vs placebo	III	797 642 resected	II–IIIB	MPR: 30.2% vs 11.0% pCR: 18.1% vs 4.0%	36 months OS: 71% vs 64% (HR 0.72) mEFS: 47.2 vs 18.3 months (HR 0.59)
CheckMate 77T Cascone et al.^49,50^	Nivolumab vs placebo	III	461 356 resected	IIA–IIIB	MPR: 35.4% vs 12.1% pCR: 25.3% vs 4.7%	30 months EFS: 61% vs 43% (HR 0.61) 30 months OS: 78% vs 72% (HR 0.85)
NADIM Provencio et al.^51,52^	Nivolumab (single arm)	II	46 41 resected	IIIA	MPR: 73.9% (34/46) pCR: 56.5% (26/46)	5 years OS: 69.3% 5 years PFS: 65.0%
NADIM II Provencio et al.^53^	Nivolumab vs placebo (2:1)	II	86 73 resected	IIIA–IIIB	MPR: 53% (30/57) vs 14% (4/29) pCR: 37% (21/57) vs 7% (2/29)	24 months OS: 85.0% vs 63.6% (HR 0.43) 24 months PFS: 67.2% vs 40.9% (HR 0.47)
AEGEAN Heymach et al.^54^	Durvalumab vs placebo	III	740 571 resected	II–IIIB	MPR: 33.3% vs 12.3% pCR: 17.2% vs 4.3%	24 months EFS: 63.3% vs 52.4% (HR 0.68)
IMpower030 Peters et al.^55^	Atezolizumab vs placebo	III	In progress	II–IIIB	N/A	N/A
RATIONALE 315 Yue et al.^56^	Tislelizumab vs placebo	III	453 363 resected	II–IIIA	MPR: 56% vs 15%	HR for EFS 0.56
NEOTORCH Lu et al.^57^	Toripalimab vs placebo	III	501 314 resected	II–IIIB	(Stage III only) MPR: 48.5% vs 8.4% pCR: 24.8% vs 1.0%	24 months EFS: 64.7% vs 38.7% (HR 0.40)
NeoCOAST-2 Cascone et al.^58^	(1) Durvalumab + Oleclumab (2) Durvalumab + Monalizumab (4) Durvalumab + datopotamab deruxtecan	II	202 186 resected	IIA–IIIB	(1) MPR: 41.9% (31/74) pCR: 20.3% (15/74) (2) MPR: 50.0% (35/70) pCR: 25.7% (18/70) (3) MPR: 63.0% (34/54) pCR: 35.2% (19/54)	–

HR, hazard ratio; ICI, immune checkpoint inhibitor; MPR, major pathological response; NSCLC, non-small-cell lung cancer; OS, overall survival; PFS, progression-free survival.

KEYNOTE-671^48^ and CheckMate 77T^49^ were two randomised, placebo-controlled, phase III trials to take this perioperative approach. In KEYNOTE-671, neoadjuvant pembrolizumab combined with cisplatin-based chemotherapy, followed by adjuvant pembrolizumab resulted in significantly improved pathological response rates (MPR 30.2% vs 11.0%, pCR 18.1% vs 4.0%), and EFS (median 47.2 vs 18.3 months) compared to placebo. At the first interim analysis, KEYNOTE-671 became the first phase III trial to demonstrate an OS benefit for chemoimmunotherapy in resectable stage III NSCLC, with 36-month OS of 71% in the pembrolizumab arm versus 64% with placebo.^59^ The HR for death was 0.72 (95% CI: 0.56–0.93, *p* = 0.0052).

CheckMate 77T followed a similar design using nivolumab.^49^ Again, substantial improvements in MPR (35.4% vs 12.1%) and pCR (25.3% vs 4.7%) were demonstrated in the immunotherapy arm. While OS is yet to be reported, 18-month EFS was significantly improved with nivolumab, at 70.2% versus 50.0% with placebo (HR 0.58, 95% CI: 0.42–0.81, *p* < 0.001), with clinical benefit seen across disease subgroups, including those with N2 involvement. Fifty-two percent of stage III patients experienced nodal downstaging, with 46% downstaging to ypN0.^60^ In updated results presented at ASCO 2025, nivolumab provided a sustained EFS benefit at 30 months (61% vs 43%; HR 0.61) and a trend towards OS improvement (78% vs 72%), though not statistically significant.^50^ Toxicity was slightly higher in the nivolumab group, with grade ⩾3 TRAEs in 32.5% of patients versus 25.2% in the placebo arm, though overall surgical feasibility was maintained.

Several additional phase III trials have adopted similar perioperative designs. In AEGEAN,^54^ durvalumab added to chemotherapy improved MPR (33.3% vs 12.3%) and EFS (HR 0.68), while RATIONALE 315^56^ and NEOTORCH^57^ reported some of the highest MPR and pCR rates to date, with tislelizumab and toripalimab, respectively.

Building on the neoadjuvant NeoCOAST trial, NeoCOAST-2 is a phase II platform study combining durvalumab with novel agents perioperatively.^58^ Of particular interest is arm 4, in which durvalumab was combined with single-agent platinum chemotherapy and datopotamab deruxtecan (TROP2-directed ADC [antibody-drug conjugate]) neoadjuvantly, followed by adjuvant durvalumab alone. MPR (63.0%) and pCR (35.2%) rates in this arm exceed those seen with standard chemoimmunotherapy combinations in previously reported trials. Although single-arm and without reporting of survival data yet, these findings suggest that integrating ADCs with immunotherapy may offer synergistic effects and represent a novel therapeutic frontier in rNSCLC.

## Immunotherapy and radiotherapy

While the current standard of care in unresectable NSCLC, established by the PACIFIC trial, is definitive chemoradiotherapy (dCRT) followed by consolidation immunotherapy for patients without progression following dCRT,^61^ the role of radiation in resectable disease is more limited. Prior to the introduction of immunotherapy, post-operative radiotherapy (PORT) after adjuvant chemotherapy was sometimes considered for patients at high risk of locoregional such as N2 disease or incomplete resection (R1/R2 disease). Although a modest benefit in terms of locoregional control is seen with PORT, this does not translate to an OS benefit.^62,63^

Perhaps of more relevance is stereotactic body radiotherapy (SABR or SBRT), which is seeing increasing use in stage I or II, node-negative tumours, particularly in patients with medical comorbidities that might preclude surgery, with results comparable to surgical resection.^64^ The benefit of immunotherapy in stage I and II NSCLC cases is less clear than in stage III, and thus far there have been conflicting results in clinical trials examining the immunotherapy in combination with SABR.

In an open-label, randomised phase II trial, Chang et al.^65^ enrolled patients (*n* = 141) with treatment-naïve stage IA–IIB, node-negative NSCLC to receive SABR +/− 4 cycles of nivolumab (480 mg 4 weekly), with the first cycle given concurrently. 4-year EFS was 77% in the nivolumab-SABR arm, compared to 53% in the SABR alone arm, with an HR in the ITT population of 0.42 (95% CI: 0.22–0.80, *p* = 0.0080). The safety profile in the nivolumab-SABR arm was felt to be acceptable, with 15% of participants experiencing grade 3 immune-related adverse events (irAEs), and no grade 3 pneumonitis or grade ⩾4 toxicities.

KEYNOTE-867, meanwhile, evaluated SBRT with or without pembrolizumab in a randomised phase III trial, but was discontinued early after an interim analysis showed no improvement in EFS (HR 0.92, 95% CI: 0.69–1.24, *p* = 0.29) and an unacceptable toxicity profile. Grade ⩾3 treatment-related adverse events occurred in 20.4% of the pembrolizumab arm (including 3.1% grade ⩾3 pneumonitis), compared to just 3.7% in the placebo arm.^66^ Additionally, there were five treatment-related deaths in the combination arm and none with SABR alone.

Ongoing trials investigating SABR-immunotherapy include PACIFIC 4 and SWOG/NRG S1914. In PACIFIC 4, a placebo-controlled, phase III trial, patients with T1–3 N0 M0 unresected NSCLC are being randomised to receive durvalumab or placebo every 4 weeks for up to 26 weeks with concurrent SBRT,^67^ a sub-cohort of patients with EGFR mutations will receive osimertinib instead of durvalumab. A slightly different approach is being taken in the ongoing SWOG/NRG S1914, a randomised phase III trial comparing SABR with or without atezolizumab in early-stage NSCLC. Patients with T1–3 N0 M0 NSCLC <7 cm, medically unfit for or declined surgery, are being randomised to SABR alone or 2 cycles of 3 weekly neoadjuvant atezolizumab, concurrent atezolizumab and SABR for cycle 3, and adjuvant atezolizumab for 6 months.^58^ Results from both trials are yet to be published.

## Discussion: Challenges and future directions

### Comparing trials, immunotherapy timing and sequencing

Heterogeneity in trial designs in the rNSCLC space makes isolating the effect of ICIs and determining the ideal timing and sequencing of immunotherapy in relation to surgery and chemotherapy very difficult. Cross-trial comparisons are, therefore, fraught with limitations and are not appropriate for making clinical decisions.

The adjuvant trials discussed above emphasise DFS improvements but do not address the potential immune-priming advantages of neoadjuvant treatment. In the CheckMate 816 trial, neoadjuvant chemoimmunotherapy demonstrated significant benefits in terms of pathological response and EFS, and has now also shown a statistically significant OS benefit. This approach has significant benefits in terms of time toxicity, cost and demands on oncology infusion capacity, as patients received just three cycles of chemoimmunotherapy prior to surgery, with no adjuvant component.

Perioperative chemoimmunotherapy, as evidenced in KEYNOTE-671, is another effective strategy with significant OS benefit. With no arm receiving neoadjuvant chemoimmunotherapy followed by placebo in the adjuvant setting; however, the trial is not designed to quantify the benefit contributed by the adjuvant component. Forde et al.^68^ presented a patient-level analysis of CheckMate 77T versus CheckMate 816 at WCLC 2024, which suggested an EFS benefit to the perioperative approach (unweighted HR 0.59 (0.38–0.92)), including those with PD-L1<1% or without pCR, though these results should be interpreted with caution as there are significant limitations to propensity score weighted analysis across trials. Prospective head-to-head trials evaluating the role of adjuvant ICI after neoadjuvant therapy will be required to directly quantify this benefit, such as in the ongoing ADOPT-Lung trial (NCT06284317).

While it may take years before these sequencing and timing questions are answered, international consensus recommendations, such as those from IASLC^69^ or ATORG,^70^ can help to guide clinicians through these difficult therapy decisions. These recommendations suggest that neoadjuvant chemoimmunotherapy is preferred (stage II) or strongly preferred (stage IIIA or IIIB) to upfront surgery in medically operable patients with EGFR/ALK WT NSCLC, regardless of PD-L1 expression. Adjuvant immunotherapy can then be considered in patients who received neoadjuvant chemoimmunotherapy.

### Patient selection and biomarkers for immunotherapy in NSCLC

While the success of the landmark trials mentioned thus far is a source for optimism in rNSCLC, it remains clear that not all patients benefit from immunotherapy. A subset of patients suitable for immunotherapy will have been cured with surgery alone, while others will exhibit tumour resistance to perioperative chemoimmunotherapy. Therefore, the identification of biomarkers to predict those that will benefit is critical to optimising the use of immunotherapy in rNSCLC.^71^

Though patients with higher PD-L1 expression or tumour mutational burden (TMB) appear to derive more benefit from ICIs, as reflected by current regulatory approvals, these biomarkers are not uniformly predictive. Indeed, a meta-analysis by Sorin et al.^72^ found that patients with PD-L1 <1% still benefit from ICIs, with pooled OS (HR 0.65 (0.54–0.79)), EFS (HR 0.59 (0.52–0.67)) and pCR rates (RR [relative risk] 5.52 [(4.25–7.15]) favouring neoadjuvant chemoimmunotherapy over chemotherapy alone. Additionally, integrating other patient-specific factors such as comorbidities, performance status and surgical feasibility further complicates the selection process.

Circulating tumour DNA (ctDNA) analysis has emerged as a promising tool for identifying high-risk patients and monitoring response to immunotherapy in rNSCLC. Patients with detectable ctDNA pre-operatively have lower RFS rates.^73^ Additionally, in NADIM II,^52^ high ctDNA baseline mutant allelic fraction (summatory mutant allelic fraction ⩾1%) at baseline and ctDNA non-clearance after neoadjuvant therapy were negatively correlated with progression-free survival (PFS; HR 4.26 (95% CI: 1.02–17.75), and HR 3.06 (0.73–12.90), respectively) and OS (HR 4.38 (0.91–21.10) and HR 6.88 (1.29–36.78), respectively), while PD-L1 and TMB status were not. An inadequate ctDNA response could therefore be used to identify patients receiving suboptimal benefit from neoadjuvant chemoimmunotherapy and inform treatment escalation. Furthermore, ctDNA can be used to detect persistent tumour cells after curative surgery, or molecular residual disease (MRD). This may help to identify those patients that derive benefit from adjuvant immunotherapy, while those with no MRD might pursue a surveillance approach.

### Role of immunotherapy in patients with actionable alterations

While patients with EGFR and ALK alterations were included in KEYNOTE-671, the trial was not powered to assess these subsets adequately. In the advanced setting, these cases typically respond poorly to ICIs with increased toxicity,^74–76^ and a molecularly targeted approach is preferred.^77–83^ An analysis by Zhou et al.^84^ found that the presence of EGFR or ALK alterations was associated with higher risk of treatment failure, shorter median time to treatment failure and reduced pathological response following neoadjuvant ICIs. Given the impressive results with targeted therapies in the adjuvant^85,86^ and now neoadjuvant^87^ settings, current data do not support the use of immunotherapy for these patients.

Beyond EGFR and ALK, increasing attention is being paid to other potentially actionable alterations, such as ROS1, RET, BRAF, MET exon 14 skipping and NTRK fusions. These alterations are rare and typically underrepresented in immunotherapy trials in rNSCLC, as much of the effort in this space has focused on perioperative or adjuvant targeted therapies. Select trials with results awaited include NAUTIKA1 (multiple, biomarker-selected perioperative targeted therapies; ClinicalTrials.gov identifier: NCT04302025), LIBRETTO-001 (cohort 7, perioperative selpercatinib in RET fusions)^88^ and GEOMETRY-N (perioperative capmatinib in MET exon 14 skipping and MET amplification).^89^

Analysis performed by Provencio et al.^90^ on samples from NADIM and NADIM II trials, and a cohort of advanced NSCLC patients (BLI-O), revealed that BRAF mutations may be an exception, as a good prognostic factor for patients receiving immunotherapy-based treatments. pCR rates were 100% in early-stage patients with BRAF mutations, compared to 44.3% in the wild-type cohort. All advanced patients with BRAF mutations were alive and without progression at time of review, compared with a median OS of 12 months for wild-type patients. Only eight patients (four early-stage, four advanced) were included, so this very small sample size limits our ability to draw definitive conclusions from this.

### Management of immunotherapy toxicity

IrAEs represent an important consideration in integrating immunotherapy into the perioperative setting. These can range from mild skin reactions or hypothyroidism to severe, life-threatening complications such as pneumonitis, colitis and myocarditis. We should also be cognisant of the fact that even ‘mild’ irAEs, such as hypothyroidism, can result in the need for life-long medication and monitoring. Managing these toxicities is particularly crucial in patients undergoing surgery, as there is a potential for severe irAEs to delay surgical timelines and complicate post-operative recovery. Despite this, in the phase III neoadjuvant and perioperative trials mentioned above, the rates of surgical cancellation ranged from 16% to 23%, similar to the rates seen in the chemotherapy only arms,^34,48,49,54,56,57^ and surgical delays were broadly similar.

Corticosteroids and other immunosuppressive agents are often required for severe irAEs, and these treatments can potentially compromise the efficacy of immunotherapy, particularly if used early and in high doses.^91^ Furthermore, the perioperative setting introduces unique considerations, as surgery-induced inflammation may exacerbate or mimic irAEs, complicating diagnosis and management. Developing standardised protocols for monitoring and managing irAEs in this context is essential. Prospective trials evaluating potentially less toxic neoadjuvant regimes and adaptive treatment strategies (e.g. intensifying or de-escalating therapy based on early imaging response or ctDNA clearance) may provide strategies to tailor treatments to patients and mitigate attrition from surgery.

### Novel therapies, technologies and new trial designs

As is so often the case, many novel treatment options are first explored in the advanced setting before being brought forward to early-stage disease. Ivonescimab, a PD-1/vascular endothelial growth factor A bispecific antibody, has shown a PFS advantage over pembrolizumab in advanced PD-L1 positive NSCLC (HARMONi-2).^92^ A phase II study by Wang et al. (NCT05247684) examining perioperative ivonescimab with or without neoadjuvant chemotherapy demonstrated very impressive MPR and pCR rates for the combination (71.8% and 43.6%, respectively) and ivonescimab alone (60% and 30%, respectively). While survival data are not yet mature, analyses have shown a robust correlation between pCR and MPR with EFS,^93^ highlighting a likely increasing role of bispecific antibodies in this space moving forward. The aforementioned NeoCOAST^24^ and NeoCOAST-2^58^ platform trials will also be pivotal for identifying synergistic immunotherapy combinations.

Future clinical trials in rNSCLC must move towards a patient-tailored approach and incorporate tools that allow for dynamic, response-informed treatment adaptation. As mentioned above, ctDNA offers a promising avenue for real-time monitoring of minimal residual disease and treatment response. Persistent or rising ctDNA post-operatively could be used to assign patients to an escalated approach, be it combination with chemotherapy, or the addition of novel immunotherapies and ADCs. A similar approach is already being assessed in Trofuse-019 (NCR06312137), a phase III trial in which patients who do not achieve pCR following neoadjuvant chemoimmunotherapy will go on to receive pembrolizumab and sacituzumab tirumotecan (a TROP2-directed ADC).^94^

In parallel, advances in artificial intelligence and machine learning hold potential to refine patient selection through integration of radiomics, histopathology and multi-omics data into predictive models.^95^ This could also identify patients unlikely to benefit from standard chemoimmunotherapy who instead require novel combinations or even non-surgical approaches.

### Real-world data

While clinical trials provide rigorous evidence of efficacy and safety, real-world data (RWD) are critical for understanding how immunotherapy performs in diverse patient populations with rNSCLC. Clinical trial participants often represent a highly selected group with specific eligibility criteria, such as good performance status and limited comorbidities. This can limit the generalisability of trial results to everyday clinical practice. RWD offers valuable insights into treatment outcomes across broader and more heterogeneous populations, including those with comorbidities, older age or rare tumour subtypes. It also allows for the evaluation of immunotherapy in settings not captured by trials, such as different healthcare systems or geographic regions.

Furthermore, RWD can help identify rare but serious adverse events and provide long-term follow-up data on OS and quality of life. Incorporating real-world evidence into clinical decision-making is essential for refining immunotherapy protocols and addressing disparities in access to care. Several RWD studies relating to immunotherapy in rNSCLC have already been reported, again with favourable MPR and EFS rates.^96–99^ As data collection and analytic tools improve, RWD will play a pivotal role in bridging the gap between clinical trials and real-world practice, ensuring the broad and equitable application of immunotherapy in rNSCLC.

## Conclusion

The introduction of immunotherapy in rNSCLC has significantly advanced the field. Landmark trials, such as CheckMate 816 and KEYNOTE-671, have led to multiple regulatory approvals and widespread clinical adoption of neoadjuvant, adjuvant and perioperative immunotherapy. These approaches, individually and in combination with platinum-doublet, have shown efficacy in addressing micrometastatic disease, reducing recurrence and extending survival, particularly in stage II and III patients.

However, significant challenges remain. Patient selection is critical, as only a subset of individuals derive substantial benefit from immunotherapy, underscoring the need for more reliable biomarkers than PD-L1 expression or TMB, such as ctDNA. Additionally, the management of irAEs, particularly in the perioperative setting, requires careful monitoring and coordinated multidisciplinary care. Questions about the optimal timing and sequencing of neoadjuvant versus adjuvant immunotherapy, as well as the utility of perioperative regimens, remain unresolved and warrant further investigation through head-to-head trials.

RWD will be instrumental in evaluating the effectiveness and safety of immunotherapy across diverse patient populations, bridging the gap between clinical trials and everyday practice. As the field continues to evolve, immunotherapy offers a pathway to improved outcomes and durable remissions in patients with rNSCLC, ultimately reshaping the standard of care for this challenging disease.

## Declarations

**Ethics approval and consent to participate:** Not applicable.

**Consent for publication:** Not applicable.

**Competing interests:** C.D.M. has no relationships to disclose. P.M.F. has received the following funding: Research Funding – AstraZeneca/MedImmune (Inst); BioNTech SE; Bristol-Myers Squibb (Inst); Novartis (Inst); Regeneron. Consulting or advisory roles – Ascendis Pharma; AstraZeneca/MedImmune; BioNTech SE; Bristol-Myers Squibb; CureVac; Daiichi Sankyo/UCB Japan; F-Star Biotechnology; Fosun Pharma; G1 Therapeutics; Genelux; Gilead Sciences; ITeos Therapeutics; Janssen; Merck; Novartis; Novocure; Regeneron; Sanofi; Synthekine; Tavotek BioTherapeutics; Teva. These disclosures are unrelated to the submitted work.

**Availability of data and materials:** Not applicable.
